# Automated annotation of rare-cell types from single-cell RNA-sequencing data through synthetic oversampling

**DOI:** 10.1186/s12859-021-04469-x

**Published:** 2021-11-19

**Authors:** Saptarshi Bej, Anne-Marie Galow, Robert David, Markus Wolfien, Olaf Wolkenhauer

**Affiliations:** 1 Department of Systems Biology and Bioinformatics, University of Rostock, 18057 Rostock, Germany; 2 Leibniz-Institute for Food Systems Biology, Technical University of Munich, 85354 Freising, Germany; 3 Institute of Genome Biology, Research Institute for Farm Animal Biology, 18196 Dummerstorf, Germany; 4 Department of Cardiac Surgery, Rostock University Medical Centre, 18057 Rostock, Germany; 5 Department of Life, Light and Matter, University of Rostock, 18059 Rostock, Germany; 6 Stellenbosch Institute of Advanced Study, Stellenbosch University, Stellenbosch, 7602 South Africa

**Keywords:** Single-cell RNA-sequencing, Imbalanced datasets, Rare cell type detection, LoRAS algorithm, Automated cell annotation

## Abstract

**Background:**

The research landscape of single-cell and single-nuclei RNA-sequencing is evolving rapidly. In particular, the area for the detection of rare cells was highly facilitated by this technology. However, an automated, unbiased, and accurate annotation of rare subpopulations is challenging. Once rare cells are identified in one dataset, it is usually necessary to generate further specific datasets to enrich the analysis (e.g., with samples from other tissues). From a machine learning perspective, the challenge arises from the fact that rare-cell subpopulations constitute an imbalanced classification problem. We here introduce a Machine Learning (ML)-based oversampling method that uses gene expression counts of already identified rare cells as an input to generate synthetic cells to then identify similar (rare) cells in other publicly available experiments. We utilize single-cell synthetic oversampling (sc-SynO), which is based on the Localized Random Affine Shadowsampling (LoRAS) algorithm. The algorithm corrects for the overall imbalance ratio of the minority and majority class.

**Results:**

We demonstrate the effectiveness of our method for three independent use cases, each consisting of already published datasets. The first use case identifies cardiac glial cells in snRNA-Seq data (17 nuclei out of 8635). This use case was designed to take a larger imbalance ratio (~1 to 500) into account and only uses single-nuclei data. The second use case was designed to jointly use snRNA-Seq data and scRNA-Seq on a lower imbalance ratio (~1 to 26) for the training step to likewise investigate the potential of the algorithm to consider both single-cell capture procedures and the impact of “less” rare-cell types. The third dataset refers to the murine data of the Allen Brain Atlas, including more than 1 million cells. For validation purposes only, all datasets have also been analyzed traditionally using common data analysis approaches, such as the Seurat workflow.

**Conclusions:**

In comparison to baseline testing without oversampling, our approach identifies rare-cells with a robust precision-recall balance, including a high accuracy and low false positive detection rate. A practical benefit of our algorithm is that it can be readily implemented in other and existing workflows. The code basis in R and Python is publicly available at FairdomHub, as well as GitHub, and can easily be transferred to identify other rare-cell types.

## Background

Single-cell RNA-sequencing (scRNA-Seq), as well as single-nuclei RNA-sequencing (snRNA-Seq), open up a transcriptome-wide gene expression measurement at single-cell level, enabling cell type cluster identification, the arrangement of populations of cells according to novel hierarchies, and the identification of cells transitioning between individual states [1]. This facilitates the investigation of underlying structures in tissue, organism development, diseases, as well as the identification of unique subpopulations in cell populations that were so far perceived as homogeneous.

### Using the single-cell technology for the identification of rare cells

**Fig. 1:**
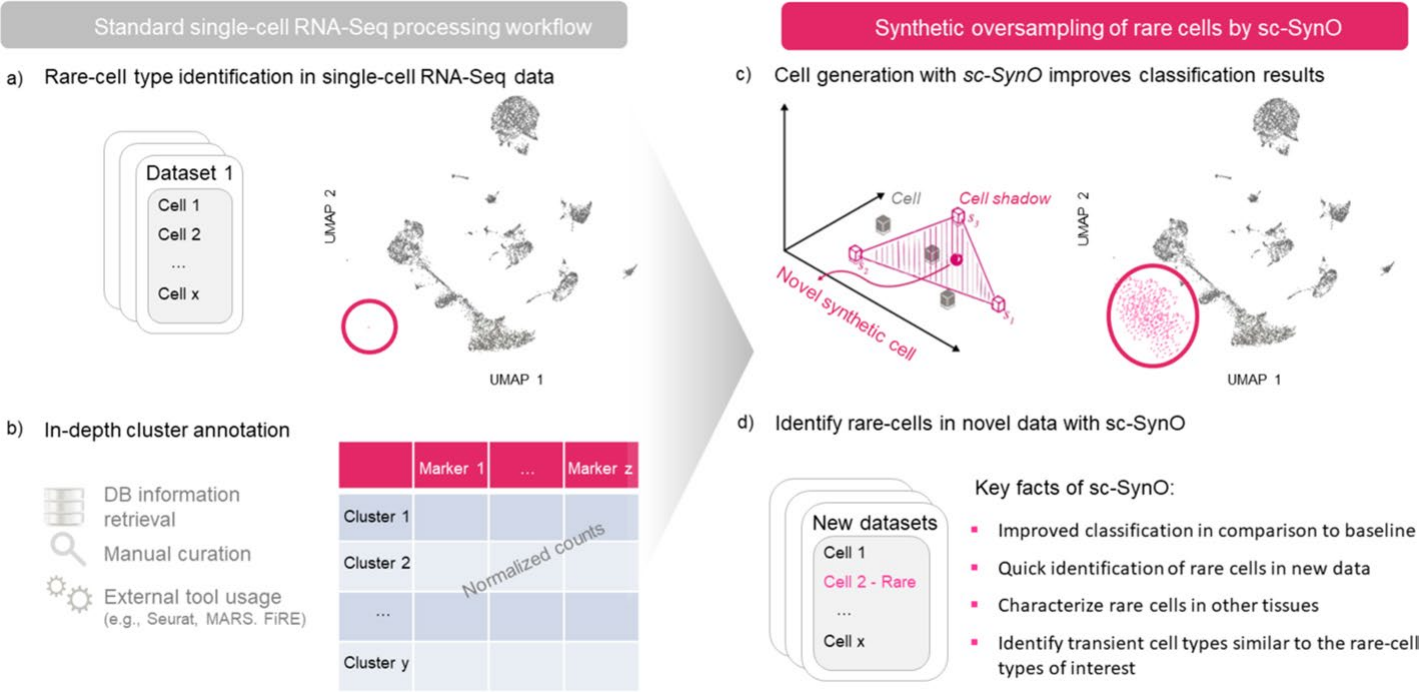
Visualization of the workflow, demonstrating a step-by-step explanation for a sc-SynO analysis. **a** Several or one snRNA-Seq or scRNA-Seq fastq datasets can be used as an input. Here, we identify our cell population of interest and provide raw or normalized read counts of this specific population. This can be done with any single-cell analysis workflow, e.g., Seurat. **b** Further information are extracted for cluster annotation that serve as improved input for the subsequent training with sc-SynO. **c** Based on the data input, we utilize the underlying LoRAS [8] synthetic oversampling algorithm of sc-SynO to generate new cells around the former origin of cells to increase the size of the minority sample. **d** The trained Machine Learning classifier is validated on the trained, pre-annotated dataset to evaluate the performance metrics of the actual model. The sc-SynO model is now ready to identify the learned rare-cell type in novel data. This figure was solely created by the authors

Classifying cells into cell types or states is essential for many biological analyses [2]. For example, investigating gene expression changes within a cell type or cell subpopulation can be of high interest across different biological conditions, time-points, or in patient samples. To be able to compare these different cell types, reliable reference systems, especially in sparse-cell states, are necessary. However, the lack of markers for rare-cell types motivates the use of unsupervised clustering approaches. Method development for such unsupervised clustering of cells has already reached a certain level of maturity [3–5]. Furthermore, many studies are interested in specialized cells (e.g., cancer cells, cardiac pacemaker cells) with an occurrence of less than 1 in 1000. The identification of such clusters, solely based on unsupervised clustering of a single dataset, remains very challenging [6]. For this reason, almost every cell clustering characterization approach is driven by manual cluster annotation, which is time-consuming and involves a bias of the annotating domain expert, thus limiting the reproducibility of results (Fig. 1). One possible solution requires a so-called cell atlas, as a curated reference system that systematically captures cell types and states, either tissue specific or across different tissues [7].

**Fig. 2:**
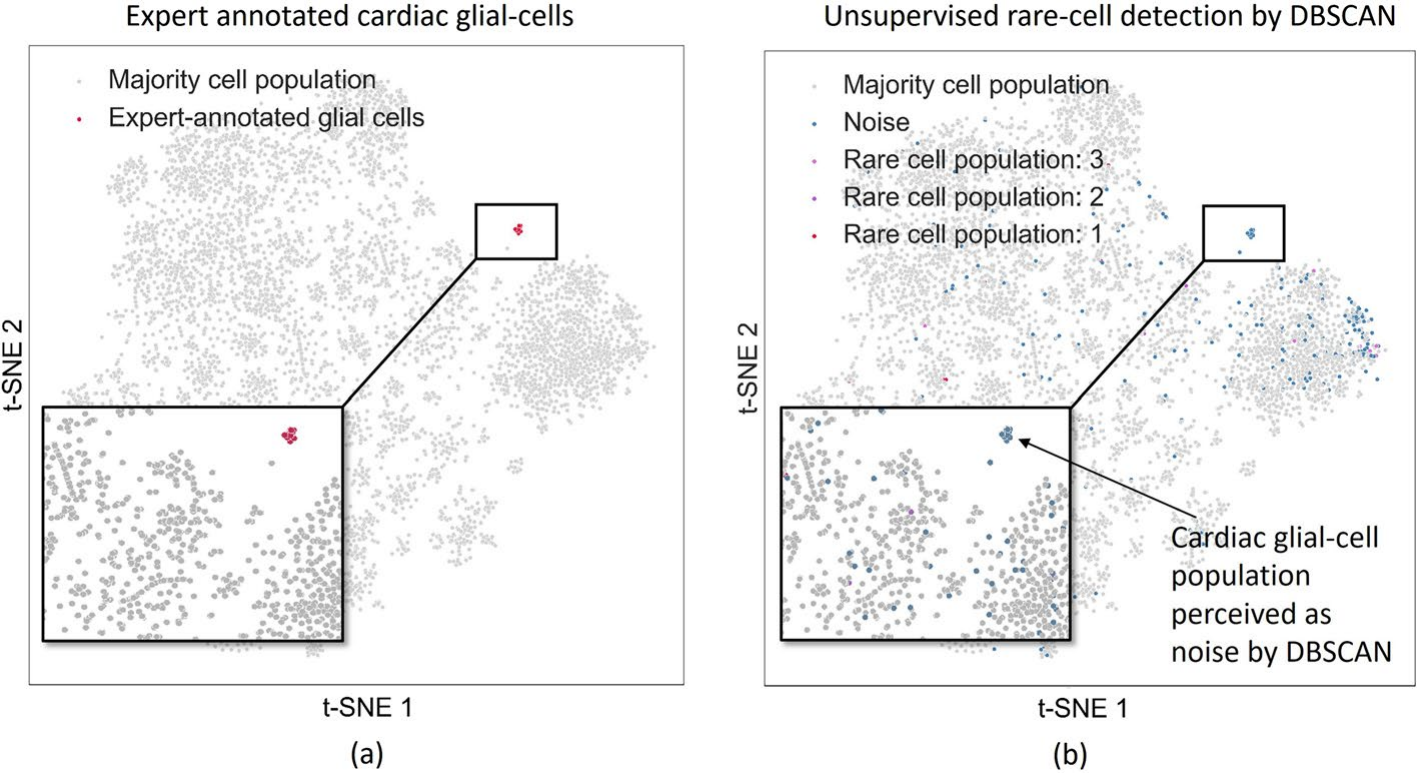
**a** Figure showing expert annotated rare cardiac glial cell population. **b** Figure showing rare-cells populations detected by the DBSCAN clustering algorithm used as a part of the GiniClust approach

It is noteworthy that there is a class of algorithms developed over recent years that are dedicated to discovery of rare-cell types. Some examples of these are RaceID [9], GiniClust [10], and FIRE [6]. However, the predictions of these techniques rely on mostly unsupervised clustering algorithms. Thus, there is always a degree of uncertainty associated with such approaches. An alternative approach to annotate rare-cells from scRNA-Seq data would be a supervised approach. If there is an expert annotated rare-cell population in a scRNA-Seq dataset, we propose to train an ML algorithm that can detect similar cells in novel datasets arising from future experiments or unseen datasets from data repositories, such as Arrayexpress or SRA. This will enable users to customize a supervised model as-per a satisfactory annotation instead of relying on unsupervised clustering methods or a complete time-consuming reanalysis of the novel data. To justify our claim, we devise a simple motivating experiment. For the experiment, we use a snRNA-Seq dataset with 8635 nuclei, out of which 17 are expert annotated as glial cells. We used the top 50 pre-selected markers for the glial-cells. Using such data, we employed the GiniClust approach to look for rare cell populations in the data in an unsupervised manner. We first calculated the Gini-index for the top 50 markers and normalized them. Then we chose the top 15 markers among them and employed the DBSCAN algorithm on the data restricted to these top 15 markers with a high Gini-index. The eps value for the DBSCAN algorithm was chosen to be 0.5 and the minimum size of clusters was chosen to be 5. Interestingly, we observed that with such a setup, the expert-annotated cluster for Glial cells was perceived as noise by the DBSCAN algorithm. We demonstrate this in Fig. 2. Assuming that a reliable annotation for a rare-cell type is available, a supervised learning approach could thus be an alternative to automatically annotate such rare-cell types in unseen datasets than an unsupervised rare-cell discovery approach. Motivated by this, in this article, we focus on a supervised machine learning approach that can be reliably used to automatically annotate rare cells on an unseen dataset based on its learning experience on a previously annotated dataset.

Here, we show how the limitation of identifying already annotated rare-cell types in newly generated scRNA-Seq data can be overcome, by using a single-cell synthetic oversampling approach (sc-SynO). sc-SynO uses the LoRAS oversampling algorithm along with a cell-type-specific marker/feature selection step. The LoRAS algorithm generates synthetic samples from convex combinations of multiple shadowsamples generated from the rare cell types. The shadowsamples are obtained by adding Gaussian noise to features representing the rare cells. The convex combination can be intuitively thought of as a weighted average of the shadowsamples [8]. For more details about the algorithm and its sample generation, we refer to the Methods section. sc-SynO is able to automatically annotate and thereby identify rare-cell types in an unbiased and precise manner in novel data.

### Using machine learning algorithms to generate cell types *in silico*

Machine learning (ML) algorithms are widely used to deal with classification problems and, thus, are used here to automate the annotation of rare-cell types from single-cell or nuclei RNA-Seq data. However, the scarce number of these cells within samples (less than 1 out of 1000 cells) often results in highly imbalanced data. An imbalanced dataset is a type of dataset where one or more classes have a significantly less number of samples compared to other classes (e.g., sinus node cells in the heart, cancer cells in the blood). A class having such a low number (minority class) is difficult to detect for unsupervised clustering approaches or classification algorithms in general [6]. The reason behind this is the inability of ML algorithms to perceive or learn underlying patterns from the minority class due to the scarcity of samples and thereby failure of these algorithms to classify them properly [11].

To overcome the problem of imbalance, oversampling techniques have been an area of research in the field of ML for more than a decade. Among several approaches proposed to deal with such issues is the approach of synthetic oversampling [12]. The philosophy of generating synthetic samples is to impute minority class instances, here cell types, in an attempt to enhance the capacity of an ML algorithm to learn. The idea of oversampling is thus commonly used to re-balance the classes [11].

In our study, we compared and benchmarked the ML-based annotation of rare cells with no oversampling against our own approach sc-SynO, which is based on our recently developed **LoRAS** algorithm applied to single-cell data [8]. Due to our knowledge, this is the first time that an oversampling approach is applied to single-cell RNA-Seq data for rare-cell detection improvement. The workflow can be obtained in Fig. 1 and is available on GitHub (https://github.com/COSPOV/sc-SynO). For more details, please see the Methods section.

## Results

### Use case preparation

To evaluate the potential of sc-SynO to precisely annotate cell populations in newly generated data, we demonstrate three use cases by utilizing already published single-cell and nuclei RNA-Seq datasets. Normalized read counts were processed with Seurat [13] (any other normalization method like SCT is also applicable). These are then used as an input to generate the synthetic samples and train the different ML classifiers. In addition, we tested the influence of using all transcripts for a classification, or only the top 20, 50, or 100 pre-selected ones (basic feature selection functions of Seurat were used [logistic regression, t-test, roc]). This helps us to investigate the influence of further downstream information obtained from standard feature selection workflows that are usually applied during scRNA-Seq analysis. If specific markers for a rare-cell type are known based on further external sources (e.g., databases, experiments, more advanced ML-based selection approaches), these can be also utilized as input features for sc-SynO.

The first use case identifies cardiac glial cells in snRNA-Seq data (17 nuclei out of 8635) [14], which were used as a training set. The trained sc-SynO ML-classifier was subsequently applied to independently generated snRNA-Seq data sets of Wolfien et al. [15] and Vidal et al. [16] to automatically detect the cardiac glial cells (Glial cells). This use case was designed to take a larger imbalance ratio (~1 to 500) into account and only uses single-nuclei data.

In contrast to this, the second use case was designed to jointly use snRNA-Seq data and scRNA-Seq on a lower imbalance ratio (~1 to 26) for the training step to likewise investigate the potential of the algorithm to consider both single-cell capture procedures at the same time and the impact of “less” rare-cell types. In particular, studies of Galow et al. [17] (snRNA-Seq) and Linscheid et al. [18] (scRNA-Seq) were used to identify prolifertive cardiomyocytes (Prl cardio).

**Fig. 3:**
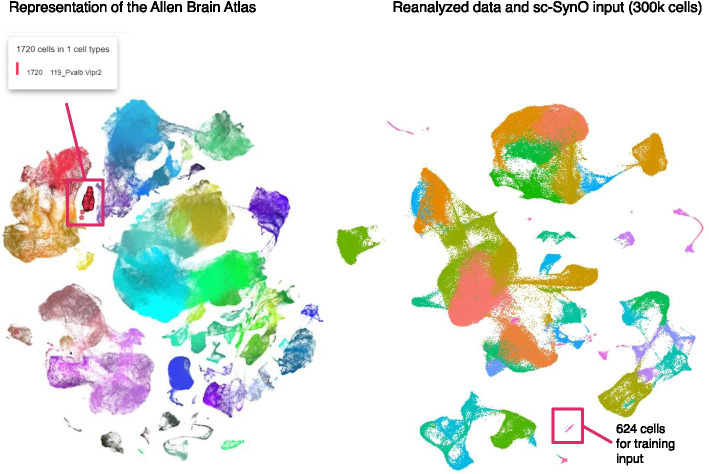
Comparison of the Allen Brain Atlas mice data of the whole dataset from (https://celltypes.brain-map.org/) and our reanalysis. The 119_Pvalb Vipr2 cluster, consisting in total of 1720 cells, was chosen as a rare-cell type of interest. The sc-SynO input was 624 cells of this cell type obtained from the first 300,000 cells in the data

The third data set refers to the murine data of the Allen Brain Atlas (https://celltypes.brain-map.org/), which serves as an example for a large dataset. Here, the expression of the first 300,000 cells have been used as a model training input to identify cells in the 119_Pvalb Vipr2 cluster (Fig. 3). Only models based on previously selected features were used for a downstream comparison because training on such a large dataset including all available transcriptomic features demands excessive computational resources. The validation of the trained model was performed on additional 300,000 cells of the murine Allen Brain Atlas dataset.

For validation purposes only, all datasets have also been analyzed traditionally using common data analysis approaches, such as the Seurat workflow, as already described elsewhere [15]. These additional experiments have been conducted to manually evaluate the identified cells from sc-SynO via traditional unsupervized clustering. The computational scripts for data pre-processing in R and sc-SynO model generation in Python can be retrieved from our FairdomHub instance (https://fairdomhub.org/assays/1368) and GitHub (https://github.com/COSPOV/sc-SynO). Key statistics, such as the imbalance ratio (Imb. ratio), number of minority samples, cross-validation folds, and oversampling neighbors of the use cases, are presented in Table 1.

**Table 1:** Key statistics of the datasets used during this study

Dataset	Imb. ratio	Minority samples	CV folds	Oversampling nbd
Glial cells	506.94	17	3	3
Prl cardio	26.21	625	10	30
Brain atlas	348.5	624	10	30

The column ’Oversampling nbd’ shows the number of nearest neighbours considered for each minority class data points to generate synthetic samples

Based on analyzed data of all three use cases, our study shows that the pre-selection of features (e.g., marker gene identification via Seurat or ML-based or manually driven) is an important pre-processing step for rare-cell type detection. Pre-selection of features not only results in faster classification models, but also produces more reliable results than using all possible features. In Table 2 we show the comparison of runtimes for several pre-selection scenarios using a knn model. We observe a much higher runtime without pre-selection of features. Moreover, the performance of the predictive model on both validation datasets without pre-selection is highly unreliable. In validation dataset VD1 and VD2 there are only five and three cardiac glial cells respectively as per expert annotations. In contrast, pre-selection of features based on prior marker identification per cluster yields much more accurate results [14, 16]. Without pre-selection the predictive model uses a large amount of features leading to an overfitted model. For example, without feature selection the predicted number of glial cells in VD1 is 0 without any oversampling and 2423 using sc-SynO. For this reason, we recommend using our workflow based on pre-selected features obtained from manually curated feature selection methods in our in-depth comparisons.

**Table 2:** Table showing comparisons among several feature pre-selection scenarios in terms of run time and efficiency in detection of glial cells for two different validation datasets (VD 1 & 2)

Dataset	Pre-selection	Pre-processing	Data generation	Training	Detected/Actual cells
VD1	All features	None	–	11 min 56s	0/5
VD2	All features	None	–	2 min 19s	0/3
VD1	All features	sc-SynO	4 min 3s	28 min 24s	2423/5
VD2	All features	sc-SynO	4 min 19s	5 min 8s	299/3
VD1	50 features	None	–	2.4 s	4
VD2	50 features	None	–	408 ms	2
VD1	50 features	sc-SynO	1.23 s	1.94 s	5/5
VD2	50 features	sc-SynO	1.12 s	484 ms	3/3
VD1	20 features	None	–	706 ms	4
VD2	20 features	None	–	240 ms	1
VD1	20 features	sc-SynO	1.12 s	679 ms	6/5
VD2	20 features	sc-SynO	1.11 s	236 ms	3/3

For simplicity, we have chosen the in-build marker gene identification methods (logistic regression, t-test, roc) from Seurat as a feature input in sc-SynO. Other commonly available methods, such as Random Forest or any other feature selection methods to derive important transcripts for the rare-cell type of interest, are also suitable. Analyses of population-specific marker genes are commonly performed for all single-cell pipelines and can contribute to the biological explainability of the model. For this reason, we have shown different case studies with different numbers of pre-selected features (e.g., 20, 50, and 100).

However, we deliberately refrain from recommending a fixed number of features for using sc-SynO because every dataset has different characteristics, and it is a common practice to tune model parameters in a dataset-specific way in machine learning. To fully understand the specific sc-SynO synthetic cell generation of a dataset, users should test different parameters, including the best amount of features by using methods, such as random grid search. Here, our results show that even with less than 100 features, the models are able to detect rare cells successfully.

**Fig. 4:**
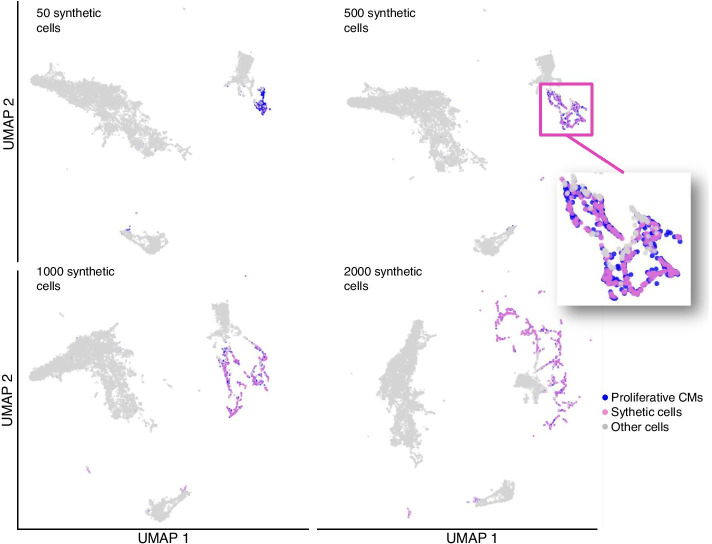
Figure showing a comparison between the distribution of synthetic cells (purple) generated by sc-SynO and original input cells (blue) for model training. The LoRAS algorithm creates synthetic cells by generating convex combinations of Gaussian noise added to rare-cell samples. This can be thought of as a random weighted average of multiple cells. In this figure, we highlight the generated synthetic and real cells to enable a visual comparison

In general, we observed that our synthetic cells were generated close to the original minority class data using UMAP visualizations (Fig. 4). To show the newly introduced cells by sc-SynO, we highlighted the generated cells for the proliferative CM cluster (50, 500, 1000, and 2000 cells respectively) and the original cells. We observed that the increased amount of *in silico* generated cells result in a stretch of the cell cluster because with larger amounts of synthetic cells the space stretches to maintain the assumption of uniformity of data distribution in UMAP.

**Fig. 5:**
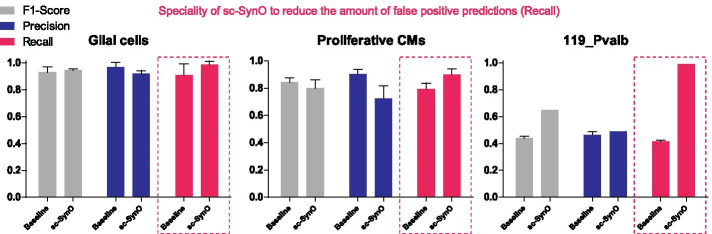
Comparison of the baseline classification and sc-SynO visualized as mean outcome for the used quality parameter: F1-Score (grey), Precision (blue), and Recall (purple). Detailed results can be obtained in Table 3. We observe that in every case, sc-SynO improves the recall compared to the Baseline model (see dotted boxes in the figure). This ensures that oversampling with sc-SynO improves the detection rate of rare-cell types (recall of the classification). Five-fold cross validation is repeated five times with five different shuffles of the dataset, which is shown as error bars to represent the variance of the performance measures over five readings

The results for all model training use cases, including pre-specified cellular markers and 5-fold stratified cross validation, are presented in Fig. 5 and more detailed in Table 3.

**Table 3:** Table showing F1-Scores/Precision/Recall for sc-SynO against baseline classification for our two ML classifiers (lr and knn) and for several numbers of pre-selected features (Marker genes). 119_Pvalb represents a small subpopulation of the Allen Brain atlas

Dataset	ML	Features	Baseline	sc-SynO
Glial cells	lr	20	.96/.94/1	.96/.94/1
Glial cells	knn	20	.97/1/.95	.94/.90/1
Glial cells	lr	50	.90/.94/.88	.94/.94/.95
Glial cells	knn	50	.89/1/.81	.94/.90/1
Prl cardio	lr	20	.80/.87/.74	.72/.62/.86
Prl cardio	knn	20	.86/.91/.81	.85/.79/.92
Prl cardio	lr	100	.86/.88/.84	.84/.81/.87
Prl cardio	knn	100	.86/.95/.79	.79/.68/.95
119_Pvalb	lr	50	.43/.45/.41	.65/.49/.99
119_Pvalb	lr	100	.45/.48/.42	.65/.49/.99

#### ML model description

For our benchmark study, we chose the k-nearest neighbors (knn) and logistic regression model (lr) as our ML classifiers. The reason behind choosing knn is that this model is known to perform well for imbalanced datasets, especially while using oversampling algorithms [19]. We also used the lr model because we observed that the effectiveness of the model in other benchmarking studies using different imbalanced datasets is performing well jointly with the LoRAS oversampling algorithm [8]. The knn model was used with $k=30$ as parameter value. After oversampling, there are almost equal data points in the majority and the minority class. For the knn classifier model, we choose a *k* value of thirty to ensure that the classifier’s decision is made on a statistically significant number of samples. The lr model was used with default parameter settings.

Given the proliferative cardiomyocyte dataset, for every ML model, we use a $5\times 10$-fold stratified cross-validation framework to judge model performances. For the cardiac glial cell dataset, due to the extremely small minority class of only 17 cells, we used a $5\times 3$-fold stratified cross-validation. First, we shuffle the dataset randomly. We divide the dataset into *k*-folds depending on the dataset as described above. The folds are kept distinct, maintaining approximately the same imbalance ratio in each fold. After we train and test our models using stratified cross validation, we identify an appropriate model for a given dataset based on the F1-score, precision, and recall. The selected model is then trained over the whole dataset and is then used to detect rare cells in two corresponding validation datasets.

### sc-SynO can detect extremely rare glial cells

#### Training data

For the cardiac glial cell dataset, all models except for the knn-Baseline model produce an F1-Score of more than 90 percent. For the lr model with 20 features, we observe that the recall is 1 irrespective of the model used. The reason behind the superior performance of all models in this case is that, even though the glial cell cluster is extremely rare, it is also very well separated from the rest of the clusters, making it easy for machine learning models to detect cells.

#### Validation

**Fig. 6:**
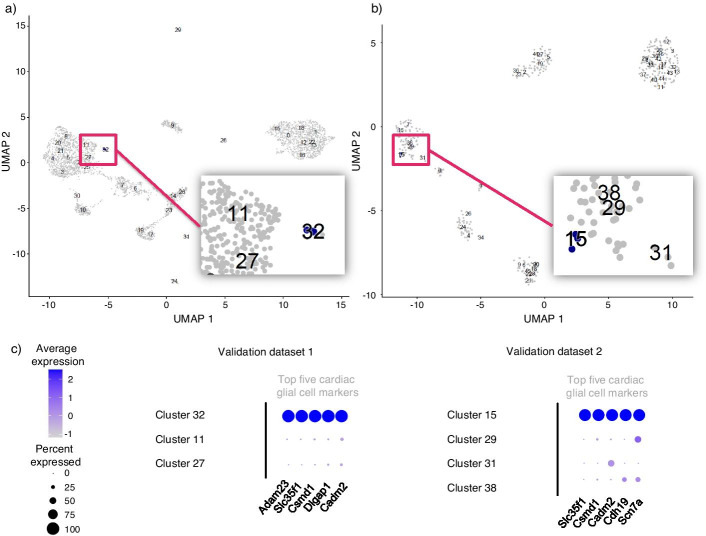
Validation of the sc-SynO model for the first use case of cardiac glial cell annotation. **a** UMAP representation of the manually clustered Bl6 dataset of Wolfien et al. [15]. Predicted cells of sc-SynO are highlighted in blue, cells not chosen are grey. **b** UMAP representation of the manually clustered dataset of Vidal et al. [16]. Precicted cells of sc-SynO are highlighted in blue, cells not chosen are grey. **c** Average expression of the respective top five cardiac glial cell marker genes for both validation sets, including the predicted clusters and those in proximity

We tested the baseline case (without oversampling) against sc-SynO using the lr model with 20 features, which was trained on snRNA-Seq normalized read count data of two independent snRNA-Seq datasets. Both, the baseline model and sc-SynO, identified four out of five cardiac glial cells in the first validation set of Wolfien et al. [14] (Fig. 6a). For the second validation dataset from Vidal et al. [16] (Fig. 6b) sc-SynO was able to detect three out of three glial cells, whereas, the baseline model was able to detect only one. Figure 6c shows the average gene expression of particular cardiac glial cell markers that are highly expressed in the identified clusters and weakly in other clusters. Although sc-SynO was effective in finding rare cells, as we have observed in this case study, the case study itself does not deterministically prove the advantage of oversampling over the baseline case. The reason behind this is as we have discussed before that the cluster of the glial cell is already well-separated within the dataset. That is why, to appreciate the effectiveness of our tool, we provide the next case study on proliferative cardiomyocyte detection. Although this cell type is not as rare as the glial cells, it is a transient cell type that is not well separated from the neighboring cluster and, thus, can appropriately show the variation in performances of the considered models.

### Sc-SynO achieves a low false negative rate for the identification of proliferative cardiomyocytes

#### Training data

**Fig. 7:**
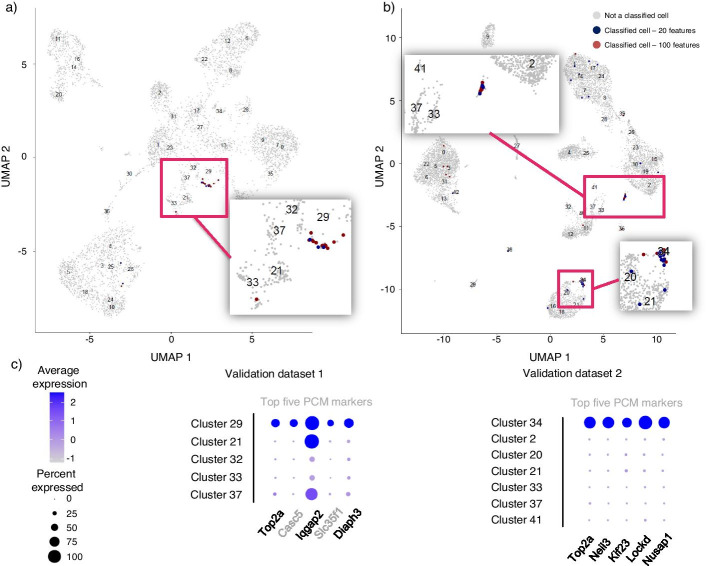
Validation of the sc-SynO model for the second use case of proliferative cardiomyocyte annotation. **a** UMAP representation of the manually clustered single-nuclei dataset of Linscheid et al. [18]. Predicted cells of sc-SynO are highlighted in blue (based on top 20 selected features in the training model), red (based on top 100 selected features in the training model) cells not chosen are grey. **b** UMAP representation of the manually clustered dataset of Vidal et al. [16]. Predicted cells of sc-SynO are highlighted in blue (based on top 20 selected features in the training model), red (based on top 100 selected features in the training model) cells not chosen are grey. **c** Average expression of the respective top five proliferative cardiomyocyte marker genes for both validation sets, including the predicted clusters and those in proximity

For the proliferative cardiomyocytes dataset, we notice that the performance of the classifiers clearly improves with including more marker genes as features (Fig. 7a, b). Note that, for all baseline models in this case, the precision is quite high. In contrast, the recall in turn is low. Due to the high precision, a high F1-Score is also maintained for the baseline models. However, given that the goal is to detect rare cells, a low recall means that the baseline models are not very effective to execute this task, even though they produce low false negatives. Interestingly, sc-SynO in turn, improves the recall, which facilitates the detection of rare cells, compromising on the precision of the classifiers. Clearly, in this case, the knn classifiers produce a higher recall and F1-score compared to the lr classifiers. With 20 features using knn for sc-SynO an improved recall to $92\%$ can be observed, while the F1-Score remains comparable to baseline.

#### Validation

We applied the baseline model and the sc-SynO algorithm on the two validation datasets for proliferative cardiomyocytes, using the knn classifier with 20 features. While with sc-SynO we were able to identify 10 out of 11 cells, the baseline case could not detect any cells from the first validation dataset. Interestingly, the model using the top 100 genes identifies 48 cells, including all 9 cells from the top 20 model, which may imply that this higher set of transcripts can detect a larger, yet similar, set of cells that are closely related to the cells of investigation. Since the second use case was about a transient cell type, the assigned cells of the model might indicate related cells that have already been or closely to enter the actual state of a proliferative cardiomyocyte. The second validation set assigned 40 cells out of 67 correctly (top 20 features). By using 100 features, the amount of correctly assigned cells increased further. In both cases, the capability of the baseline model to detect numerous cells was limited (None with 20 features and only 29 with 100 features).

### Sc-SynO can detect rare-cell populations from large-scale datasets

#### Training data

To test the effectiveness of sc-SynO on large-scale datasets, we performed our approach on the murine data of the Allen Brain Atlas (https://celltypes.brain-map.org/), which includes more than 1,000,000 cells. In our third case study, we chose a small population of cells (119_Pvalb Vipr2, see Fig. 3) and tested the effectiveness of baseline classifiers and sc-SynO on detecting this rare cell-population. The rare-cell population of interest has an imbalance ratio of 348.5. Since the dataset was very large, we performed a pilot study to notice that lr is not only the fastest among the models, but performs significantly better than knn in this case (dns). Thus, we used only the lr model for our classification task. We noticed that sc-SynO significantly improves the classifier performances. With 20 features, the precision, recall, and F1-Score for the baseline model were 0.486, 0.424, and 0.450 respectively, while with 50 features the values were 0.456, 0.416, and 0.433. For sc-SynO, with 20 features the precision, recall and F1-Score were 0.492, 0.988, and 0.655 respectively, while with 50 features the values were 0.496, 0.990, and 0.665.

#### Validation

For validation, we tested the baseline and sc-SynO oversampled trained model for an additional 300,000 cells of the Allen Brain Atlas dataset. A manual analysis of the dataset served as ground truth and resulted in ~1,500 cells of the target cluster 119_Pvalb Vipr2. Using the top 10 features, the baseline model identified 388 cells correctly in comparison to 491 correctly assigned cells of sc-SynO (375 cells in common). In addition, sc-SynO mis-classified around 200 fewer cells in comparison to the baseline model (915 vs. 734 cells).

## Discussion

Note that, from the results, use of oversampling increases the recall or sensitivity of the classification. This means that oversampling can improve the true-positive detection rate or the rate of detection of the rare-cells in this case. For the first two case studies, where the baseline classifier can already detect the rare cell population reliably, oversampling still improves the recall. However, its real usability can be observed from the third case, where the rare-cell population is not well-separated from a similar population, which is characterized by a lower Vipr2 gene expression.

Our tool is the first oversampling approach to identify and annotate rare-cell populations from scRNA-Seq and snRNA-Seq data. The LoRAS algorithm has been benchmarked against other popular oversampling techniques like SMOTE or Borderline-SMOTE, and present LoRAS as the underlying algorithm of sc-SynO as a robust algorithm for a broad set of applications in terms of F1-score and balanced accuracy [8]. For the baseline models trained without oversampling, we observe a clear limitation on the validation datasets. Since the identification of rare cells in new unseen data is a key requirement, a high recall, as obtained from oversampling approaches, would be essential. Moreover, oversampling with sc-SynO produces comparatively balanced ML model performances on average, in the sense that, in most cases, our algorithm produces less mis-classifications on the majority class with a reasonably small compromise for mis-classifications on the minority class. This is why, we would suggest using our algorithm for rare-cell detection instead of the baseline model without oversampling.

We also investigated the similarity of the original cells in comparison to the synthetic cells. We visualized the results using UMAP plots represented in Fig. 4. We noticed that plotting the synthetic samples of the minority class data space along with the synthetic samples looks stretched. We assume this phenomenon can be explained by investigating the mathematical assumptions underlying the UMAP algorithm. Note that, the full name of UMAP is ’Uniform Manifold Approximation and Projection’. The word ’Uniform’ is very important in this context. The UMAP algorithm relies on building a neighborhood graph in the process of clustering and the basic assumption behind this construction is the *uniformly* distributed data over the whole data space, even though it is not the reality. Now what happens when we oversample on a very small population is that, within a very small volume of the data space we synthetically generate numerous data points. Whereas during clustering, we assume that they are all uniformly distributed. Since there are a lot of data points, congested in a small volume, under the assumption of uniform distribution, this volume in the space itself stretches. That is why, in the 2-D plot the distribution of synthetic data points looks stretched and spread over a lot of the space. In summary, with a higher density of points in a data subspace it will be more stretched to satisfy the assumption of uniformity. Thus, if we ignore the stretching effect caused by UMAP due to a high density of synthetic samples in a small data neighborhood, we can observe that the synthetic samples are indeed quite similar to the original minority class samples as shown in Fig. 4.

Our tool facilitates the identification of very similar cells for smaller sets of feature genes and biologically related cells for larger sets of genes. The initial clustering of the training data plays an essential role, in which we observed that smaller clusters with a distinct border to other clusters are better suited for an analysis in comparison to larger cell populations with transient borders. However, the algorithm still has high accuracies in identifying those cells, but the rate of false positive predictions likewise increases.

In comparison to other current tools, such as cscGAN [20], scANVI [21], MARS [22], FiRE [6], and ELSA [23], sc-SynO uses synthetic oversampling of previously, manually curated cell populations to identify such rare cells of interest in novel unseen data. In addition, sc-SynO is easily applicable and only requires a single, well-curated dataset of any size, including only a few cells of interest, to be able to achieve already a high predictive accuracy. In conclusion, our algorithm can be used on both scRNA-Seq and snRNA-Seq representing the underlying biological heterogeneity of the sample in an improved manner [15].

## Conclusion

sc-SynO can be seamlessly incorporated in any single-cell and single-nuclei data analysis workflow after the identification and annotation of cell populations on raw or normalized read count data and important transcripts per cluster. As a potential perspective, it might be even possible to generate synthetic cells/samples out of homogeneous bulk-RNA-Seq data by treating a single sample as one single cell that needs to be identified within a single-cell dataset. Once a rare-cell population has been identified and carefully checked by a domain expert, sc-SynO can be used on this highly curated dataset to train the specific cell type. Based on our experience with the application of the oversampling models, we would see individual models for single rare-cell types as the preferred solution rather than embedding multiple minority class cell types in one data augmentation model. Applying sc-SynO on a novel dataset to identify the same rare-cell type is magnitudes less time-consuming than manually curated data processing and annotation of scRNA-Seq data. This facilitated cell enrichment can be used for more in-depth downstream analyses with the cell type of interest, without re-analyzing all the datasets. Such a scenario can be of high interest for single-cell identification in cancer, hypothesis testing on larger cell sets, cell homology search across tissues, or further individual applications.

## Methods

### Single-cell data analysis

Typical data processing of scRNA-Seq involves alignment, quality control, normalization, confounding factor identification, dimensionality reduction, and cell-gene level analysis [24]. Alignment of the raw data was conducted by using kallisto (v.0.46.1) for use case 1 and the CellRanger Software (v.6.1.1) provided by 10x Genomics for use case 2. All investigated scRNA-Seq and snRNA-Seq fastq data files were aligned to the mm10 genome (Ensembl release 93) index, annotated via the respective GTF file, and grouped by barcodes and UMIs resulting in a feature-barcode matrix. The third use case relied on already counted files that have been publicly deployed at the Allen Brain atlas platform. Downstream analysis was performed using Seurat (v.4.0.0). After following the standard pipeline of normalization (normalization.method = LogNormalize, scale factor = 10000), finding variable features (selection.method = vst, nfeatures = 3000), scaling, and dimensionality reduction by principal-component (PC) analysis, the datasets were annotated based on descriptions that have been already published elsewhere [14, 15, 17]. In brief, to assign the underlying cell types of the generated clusters, we utilized several approaches accounting for the complexity of the dataset. Sets of well-known marker genes, as well as novel cell cluster markers recently identified by other groups working with single-nuclei data, were applied as indicated in our provided computational script [16, 18]. In addition, the top 100 transcripts per cluster and the identified cell cluster markers from the specific datasets served as reference points for the full characterization of all clusters. A detailed R script is available at GitHub (https://github.com/COSPOV/sc-SynO) and FairdomHub (https://fairdomhub.org/assays/1368).

### The LoRAS algorithm as a basis for sc-SynO

The LoRAS algorithm is designed to create a better approximation of the underlying data manifold by a rigorous modelling of the convex data space compared to pre-existing algorithms, like SMOTE and several of its already presented extensions [25, 26–28, 29]. To generate a synthetic sample, the algorithm first considers the *k*-nearest neighbors of a minority class data point from a two-dimensional embedding of the minority class achieved by using t-SNE. When there are enough data points in the minority class, this provides the algorithm a better approximation of the local data manifold for the minority class.

Once the *k*-nearest neighbors are decided for a minority class data point *p* and thereby the neighborhood of *p* is identified, Gaussian noise is added to all the data points in the neighborhood of *p*. The pseudo data points generated by the Gaussian noise are called shadowsamples. A random convex combination of multiple shadowsamples is used to create a Synthetic LoRAS sample. A mathematical explanation of the algorithm asserts that, using convex combination of multiple shadowsamples in LoRAS, can produce a better estimate of the local mean considering the synthetic samples generated in a neighborhood are random variables [8]. A brief outline of the sample generation of sc-SynO approach, as well as the resulting benefits, are shown in Fig. 1.

### Oversampling procedure

Although there are several other oversampling strategies, convex combination based oversampling can work particularly well when there are too few data points in the minority class due to a lesser chance of overfitting. For every test fold, we oversample only on the training fold, so that the test fold is completely unseen to the classifiers. We specify the most important parameter values of the oversampling model to ensure full reproducibility of our models. For the proliferative cardiomyocytes dataset having 625 minority class samples, we choose 30 of the nearest neighbours of a minority class sample, as the oversampling neighbourhood for sc-SynO. sc-SynO has some additional parameters, such as ${N_{aff}}$, ${L_{\sigma }}$, and ${N_{gen}}$, enabling a better approximation of the minority class data manifold. For the Glial cell dataset, with only 17 minority class samples, we use three of the nearest neighbours of a minority class sample, as well as the oversampling neighbourhood. The *num_afcomb* parameter is chosen to be 23 and 100 for the two cases studies of the proliferative cardiomyocytes dataset with 23 and 100 prioritized marker-genes, respectively. For the Glial cell dataset, *num_afcomb* is chosen to be 50 in both case studies. For detailed parameter values, please see the code published on FairdomHub (https://fairdomhub.org/assays/1368).

Choosing proper performance metrics are also often a challenge for imbalanced datasets. The usual performance measures, such as accuracy or area under the curve (AUC) of receiver operating characteristic (ROC) might be unreliable in this scenario [30]. In our studies, we used three performance measures, precision, recall, and F1-Score (Harmonic mean of precision and recall). Here, a high precision model indicates a low number of false negative cells, relative to the number of true-positives and a high-recall model indicates a higher proportion of true positive cells. These two measures can provide in combination a fair understanding of a classifier performance on the underlying datasets. However, in the specific case of detecting rare cells, the primary priority can be user assigned to either prefer a high recall or precision score by appropriately choosing above-mentioned parameters during the training step. The F1-Score determines how well the model is balanced in terms of precision and recall. In combination, these scores can indicate how appropriate the classification model has performed.

### Model implementation, execution, and distribution

To allow for an enhanced reusability and transparency of our analysis we provide Jupyter notebooks, which can be easily utilized to rerun our analyses or adapt our proposed algorithm to other sc/snRNA-Seq experiments. We used Python version 3.7.4. The initial code basis of LoRAS is described in Bej et al. [8] and can be accessed at GitHub (https://github.com/narek-davtyan/LoRAS). To ensure a broad and versatile use of the proposed algorithm, we performed our benchmarking study on a basic personal computer (Processor: Intel(R) i7-8550U CPU @ 1.80GHz, 4 core(s), 16 GB RAM). In addition, for the computation of the Allen Brain Atlas dataset, we utilized a CentOS Linux compute node equipped with 64 cores and 754 GB RAM.

## Data Availability

All computational scripts can be obtained from our FairdomHub instance (https://fairdomhub.org/assays/1368), or the algorithm itself (https://github.com/narek-davtyan/LoRAS), and the current integration for sc-SynO on GitHub (https://github.com/COSPOV/sc-SynO). Single-cell RNA-Seq data utilized during this study are already publicly available at the Single Cell Expression Atlas via ArrayExpress (E-MTAB-7869, E-MTAB-8751, E-MTAB-8848), the Allen Brain Atlas (https://celltypes.brain-map.org/), and GEO (GSE130710).

## References

[1] Lähnemann D, Köster J, Szczurek E (2020). Eleven grand challenges in single-cell data science. Genome Biol.

[2] Lee J, Hyeon D, Hwang D (2020). Single-cell multiomics: technologies and data analysis methods. Exp Mol Med.

[3] Duó A, Robinson M, Soneson C. A systematic performance evaluation of clustering methods for single-cell rna-seq data [version 2; peer review: 2 approved]. F1000Research 2018;7:1141. 10.12688/f1000research.15666.1 PMC6134335 30271584

[4] Freytag S, Tian L, Lönnstedt I, Ng M, Bahlo M. Comparison of clustering tools in r for medium-sized 10x genomics single-cell rna-sequencing data [version 2; peer review: 3 approved]. F1000Research, 2018;7, 1297. 10.12688/f1000research.15809.1 PMC6124389 30228881

[5] Kiselev VY, Andrews TS, Hemberg M (2019). Challenges in unsupervised clustering of single-cell rna-seq data. Nat Rev Genet.

[6] Jindal A, Gupta P, Jayadeva, Sengupta D. Discovery of rare cells from voluminous single cell expression data. Nat Commun. 2018;9:12. 10.1038/s41467-018-07234-6 PMC6226447 30413715

[7] Zhang F, Lehallier B, Schaum N, Li TQ (2018). Single-cell transcriptomics of 20 mouse organs creates a tabula muris. Nature.

[8] Bej S, Davtyan N, Wolfien M, Nassar M, Wolkenhauer O (2021). Loras: An oversampling approach for imbalanced datasets. Mach Learn.

[9] Grün D, Lyubimova A, Kester L, Wiebrands K, Basak O, Sasaki N, Clevers H, Oudenaarden AV (2015). Single-cell messenger RNA sequencing reveals rare intestinal cell types. Nature.

[10] Jiang L, Chen H, Pinello L, Yuan G. GiniClust: detecting rare cell types from single-cell gene expression data with Gini index. Genome Biol. 2016;17. 10.1186/s13059-016-1010-4 PMC4930624 27368803

[11] Santoso B, Wijayanto H, Notodiputro KA, Sartono B (2017). Synthetic over sampling methods for handling class imbalanced problems: a review. IOP Conf Ser Earth Environ Sci.

[12] Weiss G, McCarthy K, Zabar B. Cost-sensitive learning vs. sampling: Which is best for handling unbalanced classes with unequal error costs? In: DMIN, 2007;pp. 35–41.

[13] Butler A, Hoffman P, Smibert P, Papalexi E, Satija R (2018). Integrating single-cell transcriptomic data across different conditions, technologies, and species. Nat Biotechnol.

[14] Wolfien M, Galow A-M, Müller P, Bartsch M, Brunner RM, Goldammer T, Wolkenhauer O, Hoeflich A, David R (2020). Single-nucleus sequencing of an entire mammalian heart: cell type composition and velocity. Cells.

[15] Wolfien M, Galow A-M, Müller P, Bartsch M, Brunner RM, Goldammer T, Wolkenhauer O, Hoeflich A, David R (2020). Single nuclei sequencing of entire mammalian hearts: strain-dependent cell-type composition and velocity. Cardiovasc Res.

[16] Vidal R, Wagner JUG, Braeuning C, Fischer C, Patrick R, Tombor L, Muhly-Reinholz M, John D, Kliem M, Conrad T, Guimarães-Camboa N, Harvey R, Dimmeler S, Sauer S (2019). Transcriptional heterogeneity of fibroblasts is a hallmark of the aging heart. JCI Insight.

[17] Galow A-M, Wolfien M, Müller P, Bartsch M, Brunner R, Hoeflich A, Wolkenhauer O, David R, Goldammer T (2020). Integrative cluster analysis of whole hearts reveals proliferative cardiomyocytes in adult mice. Cells.

[18] Linscheid N, Logantha SJRJ, Poulsen PC, Zhang S, Schrölkamp M, Egerod KL, Thompson JJ, Kitmitto A, Galli G, Humphries MJ, Zhang H, Pers TH, Olsen JV, Boyett M, Lundby A (2019). Quantitative proteomics and single-nucleus transcriptomics of the sinus node elucidates the foundation of cardiac pacemaking. Nat Commun.

[19] Blagus R, Lusa L (2013). Smote for high-dimensional class-imbalanced data. BMC Bioinform.

[20] Marouf M, Machart P, Magruder S, Bansal V, Kilian C, Krebs C, Bonn S (2018). Realistic in silico generation and augmentation of single cell rna-seq data using generative adversarial neural networks. Nat Commun.

[21] Xu C, Lopez R, Mehlman E, Regier J, Jordan M, Yosef N (2021). Probabilistic harmonization and annotation of single-cell transcriptomics data with deep generative models. Mol Syst Biol.

[22] Brbić M, Zitnik M, Wang S, Pisco A, Altman R, Darmanis S, Leskovec J (2020). Mars: discovering novel cell types across heterogeneous single-cell experiments. Nat Methods.

[23] Wang L, Catalan F, Shamardani K, Babikir H, Diaz A (2020). Ensemble learning for classifying single-cell data and projection across reference atlases. Bioinformatics.

[24] Wolfien M, David R, Galow A-M (2021). Single-Cell RNA sequencing procedures and data analysis. Bioinformatics.

[25] Chawla NV, Bowyer KW, Hall LO, Kegelmeyer WP (2002). Smote: Synthetic minority over-sampling technique. J Artif Intell Res..

[26] Han H, Wang W-Y, Mao B-H. Borderline-smote: a new over-sampling method in imbalanced data sets learning. In: Advances in Intelligent Computing. ICIC, 2005;vol. 3644, pp. 878–887. Springer, Berlin. 10.1007/1153805_91.

[27] Nguyen HM, Cooper E, Kamei K (2011). Borderline over-sampling for imbalanced data classification. Int J Knowl Eng Soft Data Paradigms.

[28] Haibo H, Yang B, Garcia E, Shutao L. Adasyn: Adaptive synthetic sampling approach for imbalanced learning. In: 2008 IEEE international joint conference on neural networks, 2008. 10.1109/IJCNN.2008.4633969

[29] Puntumapon K, Waiyamai K. A pruning-based approach for searching precise and generalized region for synthetic minority over-sampling. In: Advances in knowledge discovery and data mining (Berlin, Heidelberg), 2012;7302, 371–382. Springer, Berlin.

[30] Saito T, Rehmsmeier M (2015). The precision-recall plot is more informative than the roc plot when evaluating binary classifiers on imbalanced datasets. PLoS ONE.

